# A qualitative exploration of barriers and facilitators to drug treatment services among people who inject drugs in West Virginia

**DOI:** 10.1186/s12954-023-00795-w

**Published:** 2023-06-01

**Authors:** Abigail K. Winiker, Kristin E. Schneider, Rebecca Hamilton White, Allison O’Rourke, Suzanne M. Grieb, Sean T. Allen

**Affiliations:** 1 Department of Health, Behavior and Society, Johns Hopkins Bloomberg School of Public Health, 624 N. Broadway St., Baltimore, MD 21205 USA; 2 Department of Mental Health, Johns Hopkins Bloomberg School of Public Health, 624 N. Broadway St., Baltimore, MD 21205 USA; 3 DC Center for AIDS Research, Department of Psychological and Brain Sciences, George Washington University, 2125 G St. NW, Washington, DC 20052 USA; 4 Center for Child and Community Health Research, Department of Pediatrics, Johns Hopkins School of Medicine, Baltimore, MD 21224 USA

**Keywords:** Opioids, Substance use treatment, Barriers, Opioid agonist medications, Rural health

## Abstract

**Background:**

The opioid overdose crisis in the USA has called for expanding access to evidence-based substance use treatment programs, yet many barriers limit the ability of people who inject drugs (PWID) to engage in these programs. Predominantly rural states have been disproportionately affected by the opioid overdose crisis while simultaneously facing diminished access to drug treatment services. The purpose of this study is to explore barriers and facilitators to engagement in drug treatment among PWID residing in a rural county in West Virginia.

**Methods:**

From June to July 2018, in-depth interviews (*n* = 21) that explored drug treatment experiences among PWID were conducted in Cabell County, West Virginia. Participants were recruited from locations frequented by PWID such as local service providers and public parks. An iterative, modified constant comparison approach was used to code and synthesize interview data.

**Results:**

Participants reported experiencing a variety of barriers to engaging in drug treatment, including low thresholds for dismissal, a lack of comprehensive support services, financial barriers, and inadequate management of withdrawal symptoms. However, participants also described several facilitators of treatment engagement and sustained recovery. These included the use of medications for opioid use disorder and supportive health care workers/program staff.

**Conclusions:**

Our findings suggest that a range of barriers exist that may limit the abilities of rural PWID to successfully access and remain engaged in drug treatment in West Virginia. Improving the public health of rural PWID populations will require expanding access to evidence-based drug treatment programs that are tailored to participants’ individual needs.

## Background

Over the past two decades, the opioid crisis in the USA has worsened, creating a sustained need for increased access to low-threshold, evidence-based substance use treatment programs [1, 2]. There are more than three million persons in the USA currently living with an opioid use disorder [3]. Yet according to the 2019 National Survey on Drug Use and Health (NSDUH), only 10.3% of all individuals ages 12 and above who had a substance use disorder received treatment in the previous year [4, 5]. A major contributing factor to low drug treatment engagement is an overall lack of program access: a 2019 geospatial analysis found that over 32% of all US counties had no opioid use disorder treatment programs [6]. Further, significant disparities in program availability exist by region, with counties in the southern and mid-central regions of the US having the lowest rates of program availability [7].

Within the USA, the Appalachian region has been disproportionately impacted by the opioid overdose crisis [8]. Spanning 13 US states, the area is characterized by high rates of nonmedical prescription drug and illicit opioid use and overdose morbidity and mortality [1, 8–10]. A 2017 report from the Appalachian Regional Commission indicated that drug overdose deaths were 37% greater in the Appalachian region than the rest of the US [10, 11]. The state of West Virginia has been particularly hard hit, reporting the highest rates of drug overdose in the country for several years [12]. In 2019, West Virginia had 52.8 overdoses per 100,000 inhabitants (compared to the national rate of 21.6/100,000) [12, 13].

An extensive body of the literature demonstrates that engagement in evidence-based drug treatment is associated with positive outcomes among people who inject drugs (PWID). Substance use disorder treatment is not standardized within the US:  Programs vary substantially in their structure, length, and composition [14]. They can be short or long term, inpatient, outpatient, or fully residential, and individual or group-level. While many require private insurance or payment out of pocket, some public funding supports substance use disorder treatment, specifically through federal/state grants and reimbursement from Medicaid, a publicly funded insurance program covering many low-income and vulnerable populations within the US [15, 16]. Treatment programs further differ in their ideological underpinnings, with some advocating an abstinence-based approach and others offering medications for opioid use disorder (MOUD).

There is strong evidence supporting the effectiveness of MOUD [17]. Three MOUD formulations have been approved by the US Food and Drug Administration: methadone, buprenorphine, and naltrexone. Methadone distribution is highly regulated and limited to specific, federally certified opioid treatment programs/clinics [18]. Patients are typically required to make daily in-person visits to these sites, where they are dosed under direct supervision by program staff. Buprenorphine is physician-prescribed and also tightly regulated, though restrictions have been relaxed in recent years to reduce access barriers in response to the COVID-19 pandemic. Buprenorphine can be offered within treatment programs, hospitals, and primary care clinics, and prescriptions typically start at a seven-day duration, increasing to 30-day doses for patients deemed clinically stable [19]. Prescribing restrictions on naltrexone are the least stringent, and this medication is often prescribed in a primary care setting. MOUD programs have been associated with a range of positive outcomes among PWID, including tempering the often-debilitating experience of opioid withdrawal [17, 20], reducing HIV and HCV infections and transmission risk behaviors, and decreasing injection drug use, risky injection practices, and all-cause and overdose mortality [21–24]. Despite the benefits of MOUD, it is difficult to access among individuals with OUD: 60% of residential treatment programs in the US do not offer MOUD, and very few (1.3%) offer a full range of available MOUD options (e.g., methadone, buprenorphine, naltrexone) [25, 26].

Drug treatment programs that do offer MOUD are often unavailable in areas of need, including many rural communities [18]. In particular, a lack of MOUD access has been documented within the Appalachian region [27–29]. With a majority of Appalachia comprised of rural areas, access to both physical and mental healthcare services is limited compared to urban areas [8, 30, 31]. Further, residents face a shortage of adequately trained substance use treatment providers and a limited number of physicians with prescribing authority for MOUD [32, 33]. While statistics related to drug treatment availability and utilization are informative, they do not speak to the lived experiences of those seeking substance use treatment, including PWID. First person accounts of challenges and enabling factors may afford nuanced insights into how to best meet the needs of this population. The purpose of this research was to qualitatively explore the barriers and facilitators to drug treatment engagement among a rural population of PWID in West Virginia.

## Methods

### Study setting and data collection

Data for this study were collected during June and July 2018 in Cabell County, West Virginia. Cabell County is a predominantly rural county that is characterized by high rates of overdose and has an estimated 2.4% population prevalence of recent (past six months) injection drug use [34–36]. Participants were recruited from the Cabell-Huntington Harm Reduction Program (operated by the Cabell-Huntington Health Department), as well as nearby areas where PWID were known to congregate, such as parks and neighborhoods. To be eligible to participate, persons were required to be a resident of Cabell County, at least 18 years of age or older, and to have recently injected drugs (past 30 days).

Interviews were guided by a semi-structured interview guide developed by the research team. Interviews were conducted by study team members in a private area. To characterize our participants, we first asked that they report their age, gender, and race/ethnicity. We subsequently asked participants a series of questions about their experiences with any form of drug treatment, including types, location, and overall thoughts and opinions about the process. Forms of drug treatment included but were not limited to: MOUD, in-patient programs, out-patient programs, and detoxification/abstinence only programs. Given the relative scarcity of drug treatment programs in the state of West Virginia, the research team sought to explore drug treatment programs broadly rather than investigate experiences with any one specific type of program. Interviews lasted approximately 45–75 min and were audio recorded and transcribed verbatim. Participants received $40 for their participation. To protect participants’ identities, all data were collected anonymously. This study was approved by the Johns Hopkins Bloomberg School of Public Health Institutional Review Board.

### Data analysis

We used an iterative, constant comparative approach for analyzing textual interview data [37]. The lead qualitative researcher developed an initial coding framework upon review of 10 transcripts. Three qualitative coders then reviewed the transcripts and revised the codebook in collaboration with the lead researcher. Once codebook development was finalized, the qualitative coders applied codes to a subset of selected transcripts. The transcripts were then reviewed to assess intercoder reliability (ICR), a means of numerically assessing agreement between two or more coders on the application of codes within a qualitative text [38]. After demonstrating satisfactory consistency (*k* ≥ 0.70) [39], qualitative coders independently coded all remaining transcripts through Atlas.ti qualitative software.

## Results

Twenty-one individuals participated in the in-depth interviews in Cabell County. Among our sample, participants ranged in age from 25 to 62 years old (mean: 37). Most participants identified as male (67%) and non-Hispanic white (95%). Eighty-six percent reported heroin as their drug of choice. All participants reported having previously engaged in at least one form of drug treatment, and most described having engaged in several drug treatment programs. For example, a 32-year-old female participant stated, “I've been in and out of treatment, Methadone, Suboxone, Subutex, inpatient rehab, outpatient rehab, I've done it all.” Similarly, a 42-year-old male explained that he had attended numerous drug treatment programs: “I've been to five drug rehabs.” Participants described several factors that served as barriers to their ability to remain engaged in drug treatment and abstain from drug use. In addition, MOUD and supportive healthcare providers facilitated successful recovery and treatment engagement.

### Barriers to sustained engagement in drug treatment and substance use cessation

#### Limited access

Participants described limited availability of and access to drug treatment programs as a primary barrier to engagement. When asked about his experience getting into treatment, one 48-year-old male highlighted that entry to programs was difficult due to long program waitlists: “Very hard. It's always hard… hard to find a place when you want. It’s always a waiting list, and it's always long.”

Participants felt that access to drug treatment was difficult due to a shortage of treatment facilities in the area, which led some individuals to seek care in programs outside of West Virginia, including in Ohio, North Carolina, and Massachusetts. One female participant who accessed a local methadone program emphasized the importance of methadone availability for people who use opioids. However, she commented that the program was poorly run and the only one available in the area:

*The methadone clinic here in Huntington, methadone is a great option especially for heroin addicts, but the way they run the place is terrible. I wish somebody would get in there and fix it, because it's the only one we have around here, [lists three town names]. Other than that, it's just the Suboxone doctors which are super hard to get into.*

The same participant went on to highlight barriers to Suboxone access due to transportation challenges, stating, “Well if you don’t live on the bus line, you have to find a ride or walk, and I mean that's the majority of the people, because there's not Suboxone doctors on the bus line really.”

#### Low thresholds for dismissal

Participants described experiencing low thresholds for dismissal from drug treatment programs, which sometimes occurred for reasons beyond their personal control. A 27-year-old male participant who reported being in a methadone program stated, “I got kicked out because I had got put in the hospital for five days. And the day after I got admitted to the hospital, I was supposed to have a drug test. I was observed. And when I missed it… because they had me in the hospital for five days, they discharged me.” This participant further reported that he was told he would have to wait at least 30 days until he could try to be readmitted to the program, leaving him without access to drug treatment during this time. Another 25-year-old female participant described being dismissed for multiple reasons over the course of her engagement in drug treatment:

*I got kicked out of [drug treatment facility #1] for acting on impulse and emotion, and then I went to [drug treatment facility #2]. The [drug treatment facility #2] didn't feel it was a very good fit for me. Then I went back to [drug treatment facility #1], and then got discharged for sneaking out at night…*

Many participants identified that their dismissal from residential drug treatment programs preceded their resumption of drug use. Among those who were in residential programs, resumption of drug use frequently occurred during the immediate post-release period, oftentimes when they were confronted with marginal housing options. A 25-year-old female participant, for example, described resuming drug use when she was forced to live in an abandoned property because she had nowhere else to stay after dismissal from a treatment program: “Don't really know why I did it, but I was in a bando [abandoned property] after being discharged from [drug treatment facility] for sneaking out, and used…”.

#### Lack of comprehensive support services

Many participants reported that upon completion or dismissal from drug treatment programs, they faced a shortage of follow-up and support services. This made sustained cessation of drug use difficult, as participants felt they did not have access to the necessary tools required to maintain sobriety. A 42-year-old female participant explained that despite expressing a strong desire to remain in drug treatment, she was discharged because staff felt she had acquired the tools needed to maintain substance use cessation. This left her without continued support to remain engaged in her recovery.

*They said they've seen that I had all the tools I needed. I just needed to apply them. And there's other people that need it [drug treatment] more than me. So they wouldn’t let me go to phase two. They said there's other people who needed it more than me.*

Another participant, a 40-year-old female, described that a lack of access to jobs and housing upon completion of detox made maintaining her sobriety challenging.

*But that's the biggest problem. There's so many that I know would go get help if they had—if there was a job available for them when they got out of a 30-day detox or a 90-day or whatever. Even if it was just a part-time three days a week cleaning the city, going around and cleaning the trash up or anything. If there was a little job for them, even minimum wage three days a week, it gives them dignity. It gives them something to look forward to. And an apartment or even just a house that they didn't have to worry about having to pay the bills right off. That's the biggest thing. That's the number one thing out here that's holding so many people back.*

#### Financial barriers

Several participants reported that while they may desire to engage in longer term treatment, they were unable to do so because of financial barriers. In particular, the longer-term recovery programs many participants sought often required a weekly payment that was unaffordable. A 40-year-old female participant, for example, explained:

*The biggest issue out there and this has been me living on the street for a year, plus being sober for six years, the biggest problem we’re having out there is so many of them want recovery and want detox and all that. But once they get to detox they have nowhere sober to go. There's nowhere—they don't have family support. They don't have friend support. The recovery houses around here are awesome, but it’s $100 a week. And when you been on the street for 2 to 3 years, you don't have $100 a week to do that.*

Participants further described that this lack of access to longer term recovery services may lead to the resumption of drug use. A male participant, for instance, explained, “…in a week and a half time they [drug treatment program] had threw me out because I didn't have the $100 they wanted. I couldn't find a job ASAP like they were wanting. So I was kind of thrown back on the streets and [resumed drug use].”

#### Insufficient withdrawal management

Many participants described experiencing painful, unmanaged withdrawal symptoms during drug treatment which served as a barrier to their willingness to remain in and/or pursue future treatment. One 37-year-old female participant described experiencing painful withdrawal symptoms during her engagement in a detox program, which led to the perception that other PWID would be unlikely to pursue treatment due to fear of unmanaged withdrawal:

*I went to two different detox programs, and they were seven to ten days detox programs. Horrible, absolutely horrible, because they didn’t want to give you anything. They wanted you to lay there and detox with nothing, absolutely nothing, and I just think that’s unfair, because it’s hard doing it, and they should be able to give us something. I mean, I really think that a lot of the addicts out here, a lot of the heroin addicts, they would be more apt to go and get clean and go into a detox program if it was a medical detox and they gave them some kind of medicine or something to help them or something like that, because most of the detox programs around here don’t, and I think it scares the crap out of people, and that’s why they don’t want to go do it, because that’s the one thing that I hear when I talk to people about it. That’s the one thing I hear. “I don’t want to do it because I know I’m going to get too sick, and they won’t give me anything.” And, with those, I just, I think it’s inhumane honestly to do people that way. … if they want to help us and they want to improve our community and improve us as citizens and stuff like that, they need to be a little bit more humane about it because I just, I completely think that’s inhumane.*

A 33-year-old male participant echoed this sentiment by explaining that the severity of his unmanaged withdrawal resulted in him leaving a treatment program: “I just—some people can go do it cold turkey, but when I went up there, they gave me medicine to do it [stop using drugs], but it just wasn’t enough. I was still going through the sickness, so I walked out.”

### Facilitators to sustained engagement in drug treatment and substance use cessation

#### Medications for opioid use disorder

Among our participants, many spoke positively about engaging in MOUD-based drug treatment programs. For example, a 27-year-old male participant noted that receiving methadone changed how he perceived his relationship with drug use:

*And then when I started going to the methadone clinic, I woke up one day and I told my girlfriend I said, “I'm done.” And she said, “You're done?” And I said, “I'm done.” I said, “Take every needle, take everything that I've got to do anything was shooting dope [and get rid of it].*

Another 34-year-old female participant explained that receiving MOUD-based treatment stopped her physiological cravings for drugs, stating: “…when I was on it for about four months, it was the best. I didn’t have—I didn't wake up thinking or wanting it [opioids]. It was like a miracle drug for me.” Similarly, a 37-year-old male participant described positive experiences with MOUDs: “…it worked, and I didn’t really think about it [using drugs] at all.”

#### Supportive healthcare providers

While most participants described a range of barriers to drug treatment engagement, several highlighted positive experiences in which they felt supported. In particular, many participants emphasized the value of warmth and open communication from care providers and support staff within the programs at which they sought care. A 32-year-old female recounted a highly positive encounter with a treatment program, describing the care she received as “great” and “personalized,” elaborating with:

*I mean you walk into the window, "Hi, blank, blank," they know you by name, everybody, every patient. You're not just a number. You see a doctor, a nurse on a daily basis, "How are you feeling?" they ask you. And groups and therapy is just so amazing. It's really nice over there.*

Another 40-year-old female participant noted experiencing a change in the quality of treatment she received from healthcare providers over time, stating: “…any of my regular doctors or anyone that I see—a lot more of them are being schooled on it [addiction], and they’re a lot more compassionate instead of being very rude like they used to be.” The experiences of these participants highlight the importance of person-centered care and mutually respectful staff, provider, and client relationships as a means of facilitating treatment engagement among PWID.

## Discussion

Through interviews with PWID in a rural county in West Virginia, we identified several barriers and facilitators to sustained engagement in drug treatment and substance use cessation. Participants described limited access to drug treatment programs and low thresholds for dismissal, often for reasons that were outside of their control. In addition, an overall lack of follow up and support services was described as a barrier to sustained recovery. Financial constraints were also reported among many participants, with prohibitive out-of-pocket expenses required for drug treatment engagement. Further, inadequate management of withdrawal was described as an impediment to engaging in drug treatment and often led to resumption of drug use. MOUD-based drug treatment programs were, however, described as being highly effective and supported persons in their recovery through the management of cravings and withdrawal symptoms. Additionally, participants expressed that having supportive relationships with their healthcare providers encouraged drug treatment engagement. Collectively, these findings demonstrate that the public health of rural PWID may be improved through the scaling up of low-threshold, evidence-based drug treatment and MOUD programs that emphasize person-centered, mutually supportive care.

It is well documented that many federal and state policies negatively affect access to evidence-based OUD treatment programs, including policies which prevent the establishment of new opioid treatment centers or limit the number of patients who can be seen at a particular site for treatment [40, 41].

Many interviewees were impacted by these policies, citing waitlists, program shortages, or the need to travel to other states due to the scarcity of local programs. This reaffirms findings from other studies which highlight that long distances and transportation barriers significantly limit access to treatment among PWID [42, 43]. Despite the many barriers in place, all 21 participants reported having been engaged in at least one form of drug treatment in the past. However, many then faced low thresholds for dismissal and inadequate support services, which exacerbated risks for resuming drug use. While we do not have sufficient data to assess every participants’ reason(s) for termination of each of these past programs, this suggests that consistent retention remains a problem among persons who access treatment.

Importantly, research has found that engagement with drug treatment programs for longer durations of time is associated with better outcomes [44, 45]. For example, a recent study found that patients who participated in shorter-term treatment programs were at a heightened risk of resuming drug use compared to persons who engaged in longer programs [46]. Further, recent evaluations of MOUD programs have found that patients who remained in longer-term, continuous drug treatment experienced improved outcomes: drug treatment duration was positively associated with drug use improvement, as measured by the frequency and type of drugs used [47–49].

One way to improve retention and substance use outcomes among PWID is the implementation of low-threshold, MOUD-based drug treatment programs that offer longer term, comprehensive services. Such programs may be particularly beneficial given the prevalence of co-occurring mental health disorders among PWID. Specifically, multidisciplinary treatment models which incorporate mental health and substance use disorder treatment are associated with reductions in opioid use, mental health symptoms, and nights in jail [50]. Delivery of wraparound services (a strengths-based model which incorporates team-based, coordinated care) to individuals in substance use treatment programs has also been shown to be positively associated with several indicators of treatment retention, including the number of treatment sessions attended and length of stay in treatment days [51].

Participants also described cost as a barrier to drug treatment engagement, underscoring the need for low-threshold access to services that is not conditioned upon one’s ability to pay. This finding is reflective of a lack of free, publicly funded OUD programming within the US, and overall insufficient health insurance coverage for drug treatment. For example, in 2018, most Medicaid programs did not cover residential treatment, and many private insurers also excluded coverage to these programs [52]. Research has also shown that 41% of adults in treatment for illicit substance use pay for it on their own, suggesting that many low income individuals face cost as a barrier to any form of substance use treatment access [52, 53]. While some treatment programs do accept private and/or public insurance, people experiencing an opioid use disorder are more likely to be uninsured, which further limits their access to care: as of 2016, only 26% of uninsured individuals with an  opioid use disorder entered any form of drug treatment [54]. Further, uninsured patients often face high out-of-pocket costs for MOUD: A 2014 Department of Health and Human Services report estimated that the median cost of buprenorphine for individuals without insurance was $539 per month, compared to only $25 per month among those with commercial insurance [55]. However, persons with public insurance often face high out of pocket expenses and additional MOUD access barriers such as prior authorization requirements [55, 56]. Eliminating these financial barriers to evidence-based drug treatment programs is a necessary step toward improving the health of individuals with OUD.

Among our participants, many reported experiencing painful withdrawal symptoms during times they engaged in drug treatment programs, particularly those which were abstinence based. Conversely, persons described MOUD-based drug treatment engagement as supportive of their efforts to achieve substance use cessation, highlighting that the medications helped them manage withdrawal symptoms. Ideally, MOUD-based drug treatment programs should be rapidly scaled up throughout rural communities. However, achieving this goal will require changes across the healthcare sector. While MOUD is recognized as the gold standard for management of persons with opioid use disorders, federal limitations on MOUD prescribing remain in place [6, 21]. For example, until recently, prescribing of buprenorphine required the completion of a time-intensive regulatory approval process known as an X-waiver through the federal government. In 2016, only 4% of US physicians were waivered to prescribe buprenorphine, and 60% of rural counties did not have a single waivered provider [57]. While the X-waiver requirement was overturned by the Substance Abuse and Mental Health Services Administration and Drug Enforcement Administration in January of 2023, providers are still limited by federal law in the number of patients they may treat at one time with buprenorphine [58, 59]. In order to address additional barriers to MOUD treatment brought about by the COVID-19 pandemic, states have seen the relaxation of some these prescribing restrictions*.* Specifically, the Drug Enforcement Administration and state regulators have enabled use of telehealth for MOUD prescribing, reduced restrictions on take-home doses of methadone, and passed laws allowing primary care providers to prescribe MOUD across some state lines [60–63]. Efforts should be made to maintain these policy changes, even with the removal of the pandemic-related state of emergency within the US. Further, telehealth can serve as an important mechanism to expand MOUD access, and additional research is warranted to assess the impact of these policies on PWID residing in rural communities.

Within the drug use treatment landscape, some persons draw a distinction between models promoting complete cessation (i.e., abstinence from use of any substances, including MOUD), and maintenance (i.e., cessation from illicit substances through the controlled use of MOUD options such as buprenorphine and methadone) [64]. However, it is important that constituencies understand the evidence-base of MOUD utilization. Persons who utilize MOUD demonstrate greater treatment retention, decreased rates of resumption of drug use, and lower risk of overdose compared to persons engaged in abstinence-based treatment [65, 66]. Our findings suggest that MOUD programs are seen as the most effective/desired option among participants, likely due in large part to the management of withdrawal symptoms and cravings.

Despite the evidence, many policymakers and treatment providers continue to favor an abstinence-based treatment model [67]. Multiple studies have demonstrated that abstinence-focused programs create significant barriers to patient access, retention, and engagement [68–70]. Substantial research has further demonstrated that many PWID report experiences of stigmatization and mistreatment by healthcare providers, which result in delayed care seeking behaviors and decreased substance use treatment engagement [71–73]. Targeted programs to reduce drug use stigma among healthcare providers have been called for as a means of reducing negative treatment of PWID and encouraging increased access to treatment for members of this population [71]. One such strategy involves comprehensive educational campaigns and training programs for providers who interact with PWID, particularly promoting integration of MOUD into medical practices and incorporating messaging that addiction is a disease, not a moral failing [20, 74]. Further, cultural competency training that stresses the importance of person-first terminology can reduce expressions of judgment toward PWID from healthcare providers [75]. Importantly, several participants highlighted that having open, respectful relationships with program staff and recovery service providers was a facilitator of substance use cessation and treatment success. This further emphasizes the importance of anti-stigma campaigns within rural communities to ensure PWID are afforded compassionate, person-centered care.

While these findings demonstrate several important barriers and facilitators to drug treatment engagement among rural PWID, they must be viewed in the context of several limitations. These data were collected in 2018 and therefore may not fully reflect the current scope of the drug treatment landscape, particularly given recent changes stemming from the COVID-19 pandemic. Further, the experience of these participants may not be transferable to PWID who reside in other communities, especially those in urban settings. The limited availability of MOUD options in the state of West Virginia led some participants to utilize detoxification or abstinence-only programs, which may be a less likely outcome among persons in communities in which a greater range of program options exist. Given that PWID who do seek treatment are typically constrained by the options available within their geographic region, it would be valuable to conduct similar work in a range of settings to explore place-based differences in treatment access and retention. Participants in this study were primarily white and male, limiting our ability to characterize differences in drug treatment experiences among women and other racial and ethnic groups. Finally, we did not collect data on participants’ education level, annual income, or other relevant indicators of socioeconomic status.

## Conclusions

From 21 in-depth interviews conducted with PWID in Cabell County, West Virginia, we found numerous barriers which limited access to and retention in drug treatment. West Virginia has been hard hit by the opioid crisis, and comprehensive solutions will be required to adequately support persons living with an opioid use disorder within the state. These first-person accounts provide important insights, underscoring opportunities to improve access to low-threshold and evidence-based drug treatment services to support substance use cessation among PWID in rural communities.

## Data Availability

The datasets used and/or analyzed during the current study are not publicly available to protect participants’ identities.

## Abbreviations

PWID: People who inject drugs
MOUD: Medication for opioid use disorder
OUD: Opioid use disorder
